# Bepridil Might be Potent Against the Coronavirus Disease 2019–related Electrical Storm in Brugada Syndrome

**DOI:** 10.19102/icrm.2022.13106

**Published:** 2022-10-15

**Authors:** Akinori Higaki

**Affiliations:** ^1^Department of Cardiology, Pulmonology, Hypertension & Nephrology, Ehime University Graduate School of Medicine, Shitsukawa, Japan

**Keywords:** Bepridil, Brugada syndrome, COVID-19, electrical storm

I read with great interest the case report by Ali and Nilsson on electrical storm in Brugada syndrome (BrS) induced by severe acute respiratory syndrome coronavirus 2 (SARS-CoV-2) infection.^1^ While experts have already made thoughtful comments on this issue,^2^ I would like to add an idea for treatment options that was not mentioned during the discussion: bepridil.

In the viral replication process, the main protease (M^pro^) of SARS-CoV-2 plays an indispensable role.^3^ Previously, Vatansever et al. reported that bepridil hydrochloride inhibits the enzymatic activity of M^pro^ in vitro, suggesting a potential antiviral effect in coronavirus disease 2019 (COVID-19).^4^ To date, bepridil has also been reported to be effective in the prevention of electrical storm in BrS.^5^ Therefore, the electrical storm reported by Ali and Nilsson might have been prevented if the patient was prescribed bepridil.

I am aware that bepridil is currently only available in limited countries (i.e., China, France, and Japan) because of its QT-prolongation side effect. However, I believe it is worthwhile to discuss possible treatment options for patients whose lives are at grave risk in the COVID-19 era.
